# Virulence, Antimicrobial Susceptibility, Molecular and Epidemiological Characteristics of a New Serotype of *Vibrio parahaemolyticus* From Diarrhea Patients

**DOI:** 10.3389/fmicb.2020.02025

**Published:** 2020-08-21

**Authors:** Qiaoyun Zhu, Xiao Chen, Yanchao Liu, Ruonan Wang, Jiayao Chen, Yu Chen

**Affiliations:** ^1^Department of Laboratory Medicine, The First Affiliated Hospital, School of Medicine, Zhejiang University, Hangzhou, China; ^2^Key Laboratory of Clinical in Vitro Diagnostic Techniques of Zhejiang Province, Hangzhou, China; ^3^Institute of Laboratory Medicine, Zhejiang University, Hangzhou, China; ^4^Central Laboratory, The First Affiliated Hospital, School of Medicine, Zhejiang University, Hangzhou, China; ^5^State Key Laboratory for Diagnosis and Treatment of Infectious Diseases, National Clinical Research Center for Infectious Diseases, Collaborative Innovation Center for Diagnosis and Treatment of Infectious Diseases, The First Affiliated Hospital, School of Medicine, Zhejiang University, Hangzhou, China; ^6^Zhoushan People’s Hospital, Zhoushan, China

**Keywords:** *Vibrio parahaemolyticus*, O4:KUT, new serotype, recA insertion, epidemiological characteristics

## Abstract

*Vibrio parahaemolyticus* is the main pathogen of food-borne diarrheal in coastal areas. Through the study of pathogen characteristics of 1870 *V. parahaemolyticus*, the isolation rate of O4:KUT had increased significantly since 2013. In this study, we analyzed virulence, antimicrobial susceptibility, molecular, and epidemiological characteristics of a new serotype named O4:KUT2. O4:KUT2 strains had *tlh*^+^ *tdh*^+^ *trh*^–^ *toxRS/*new^–^ characteristics and were prevalent during 2013–2015. The 91.5% O4:KUT2 serotype strains were resistant to ampicillin. The growth curves of O4:KUT2 strains were different with O4:K9, O4:K8, and O3:K6 serotype strains. O4:KUT2 strains belonged to ST332 where four strains had a large fragment inserted at *recA*. Compared the whole genomes of O4:KUT2 strains with O4:K9 strain which also belonged to ST322 isolated from acute diarrhea patients in Zhejiang province in 2012, no different alleles at 2249 loci was found. This finding implied that O4:KUT2 strains might be derived from O4:K9 strains. Clinical presentation of patients positive for *V. parahaemolyticus* O4:KUT2 were no significant difference with patients positive for O3:K6, although their genetic characteristics were different. The appearance and the increase of proportion of the new serotype O4:KUT2 strains was aware that we should not relax the monitoring of the pathogen spectrum of acute diarrheal patients.

## Introduction

*Vibrio parahaemolyticus* is the main pathogen of food-borne diseases in coastal areas (Klose and Satchell, 2018). Ingestion of food containing *V. parahaemolyticus* can cause food poisoning. The first case was reported in 1950, when 272 people were collectively poisoned by eating contaminated dried herring in Osaka, Japan, resulting in 20 deaths (Fujino et al., 1953). However, the outbreak of *V. parahaemolyticus* did not show obvious serotype specificity until February 1996, a large-scale food poisoning event broke out in Calcutta, India, caused by *V. parahaemolyticus* serotype O3:K6 (Okuda et al., 1997). Subsequently, O3:K6 became the highest detected serotype of *V. parahaemolyticus* in the world (Chiou et al., 2000; Ansaruzzaman et al., 2005; Martinez-Urtaza et al., 2005; de Jesús Hernández-Díaz et al., 2015). The O3:K6 strains with characteristics of *tlh*^+^ *tdh*^+^ *trh*^–^ *toxRS*/new^+^ were defined as pandemic groups (Matsumoto et al., 2000). So far, the *tlh*^+^ *tdh*^+^ *trh*^–^ *toxRS*/new^+^ genetic characteristics were found in at least 20 serotypes, including O3:K6, O4:K68, O4:K8, O1:K25, and O1:KUT (K untypeable) (Nair et al., 2007). The emergence of these pandemic characteristics was thought to be serotype transformation.

We monitored the pathogen spectrum of acute diarrheal patients in Zhejiang province since 2009. In the process of monitoring, we found that *V. parahaemolyticus* had changed significantly in the composition of serotypes since 2013. The detection rate of O4:KUT serotype had exceeded O3:K6 and became the highest in 2013. In the following years, the rate of O4:KUT remained high. To find out whether O4:KUT is one serotype or contains multiple serotypes, we selected five O4:KUT strains per region per year to sequence their whole genome. According to the analysis of K antigen gene clusters sequences between *gmhD* and *rig* (Okura et al., 2008; Chen et al., 2010), there were mainly two kinds of KUT. We named them O4:KUT-recAin and O4:KUT2. Information of O4:KUT-recAin was reported in our published article (Chen et al., 2020). In this article, O4:KUT2 was focused, including analysis of the K antigen gene clusters sequences of O4:KUT2, discussion of virulence, antimicrobial susceptibility, molecular and epidemiological characteristics, and the origin of O4:KUT2 strains.

## Materials and Methods

### *V. parahaemolyticus* Strains

About 1870 *V. parahaemolyticus* strains were isolated from acute diarrhea patients (three or more watery or loose stools per day with a duration of no more than 14 days) in six general hospitals and one children’s hospital from 2009 to 2017 in Zhejiang province (Supplementary Table S1). Strains from other regions were provided by Center for Disease Control and Prevention of Jiangsu province and Liaoning province.

Stool specimens were cultured in thiosulfate citrate bile salt sucrose agar (TCBS agar) culture medium after alkaline peptone water (APW) enrichment to isolate microorganisms. *V. parahaemolyticus* was identified by MALDI-TOF microbial mass spectrometer (Bruker, Germany) according to the instructions (Jorgensen and Pfaller, 2015).

### Serotyping

*Vibrio parahaemolyticus* strains were serotyped using agglutination tests with 11 O (lipopolysaccharide) and 65 K (capsule) antisera (Denka Seiken Ltd., Japan) according to the instructions provided with the reagents. Serotype was defined as a unique combination of O and K. OUT reffered to O untypeable while KUT referred to K untypeable. KUT strains were also test by K72, K73, K74, and K75 antisera (Denka Seiken Ltd., Japan).

### Whole-Genome Sequencing

Genomic DNA of *V. parahaemolyticus* was extracted by the Yeast/Bact Kit B (Puregene, Germany) and randomly broken by ultrasonic crusher (Covaris, United States) at length of 350 bp. The whole library was prepared by terminal repair, addition of A-tail, addition of sequencing connector, purification, PCR amplification and HiSeq PE150 sequencing (Illumina, United States) with a minimum coverage of 120-fold. The sequencing results were assembled by SOAPdenovo software (Version 2.04) and then predicted by GeneMarkS (Version 4.17). Gene Ontology (Version: May 2017), Cluster of Orthologous Group of proteins (COG), and the Kyoto Encyclopedia of Genes and Genomes (KEGG) database (Version: 2016) were used for annotating gene function.

Accession numbers of Whole-genome sequencing (WGS) in NCBI from this study were JACBKD000000000, JACBKE000000000, JACBKF000000000, JACBKG000000000, JACBKH000000000, JACBKI000000000, JACBKJ000000000, JACBKK000000000, JACBKL000000000, and JACBKM000000000.

### O4:KUT2 Strains Screening by PCR

Primers for K antigen gene cluster specific sequences of KUT2 were designed to identify O4:KUT2 strains from O4:KUT which were listed in Supplementary Table S2. The bacterial DNA template for PCR amplification was from boiling method. The specific steps were as follows: the bacterial colony size of match head was picked into 500 μl deionized water, shaken for 10 s, cracked at 100°C for 10 min, and centrifuged at 13000 rpm for 10 min. Three microliter supernatant was taken as the PCR template. The components of PCR reaction solution composed of 12.5 μl Premix Taq (Takara, Japan), 3 μl bacterial DNA, 0.5 μl forward primer (20 μmol/l), 0.5 μl reversed primer (20 μmol/l), and 8.5 μl ddH_2_O. Polymerase chain reaction (PCR) were as follows: denaturation at 94°C for 5 min, followed by 30 cycles of 94°C for 30 s, 55°C for 30 s, and 72°C for 1 min, and extension at 72°C for 10 min in ABI Veriti thermal cycler.

### Multilocus Sequence Typing

Multilocus sequence typing (MLST) was performed according to the protocols published on the website^1^ (Jolley and Maiden, 2010). Seven house-keeping genes (*dnaE, gyrB, recA, dtdS, pntA, pyrC*, and *tnaA*) were selected for PCR amplification and sequencing. Core genome multilocus sequence typing (cgMLST) including 2254 loci were selected for whole genome analysis (Gonzalez-Escalona et al., 2017).

### Detection of Genetic Characteristics

Polymerase chain reaction assays were taken to test the species-specific marker (*tlh*), hemolysin genes (*tdh* and *trh*), *orf8* and *toxRS*/new (GS-PCR). The primers were listed in Supplementary Table S2. The bacterial DNA template for PCR amplification was prepared as above. The PCR mixtures consisted of 12.5 μl Premix Taq (Takara, Japan), 3 μl bacterial DNA, 0.5 μl forward primer (20 μmol/l), 0.5 μl reversed primer (20 μmol/l), and 8.5 μl ddH_2_O. The PCR reaction parameters were as following: denaturation at 94°C for 5 min, circulation by 30 cycles with 94°C for 30 s, each gene annealing temperature (Supplementary Table S2) for 30 s and 72°C for 1 min, and extension at 72°C for 10 min in ABI Veriti thermal cycler. The type III secretion system 1 (T3SS1) genes VP1670 (*vscP*), VP1686 (*copS*), VP1689 (*vscK*), and VP1694 (*vscF*), the T3SS2α genes VP1362 (*vopB2*), VP1339 (*vscC2*), VP1335 (*vscS2*), and VP1327 (*vopT*), and the T3SS2β genes (*vscC2*, *vopB2*, *vopC*, and *vscS2*) were detected on 2% agarose gel electrophoresis after four-multiplex PCR (Jones et al., 2012).

### Antimicrobial Susceptibility Testing

Antimicrobial susceptibility testing was performed using Kirby-Bauer method by 18 kinds of conventional antibiotics (Magiorakos et al., 2012). The interpretation of susceptibility, mediation and drug resistance were referred to the latest standard recommended by M45-Ed3E standards formulated by Clinical and Laboratory Standards Institute (CLSI, 2015). *Escherichia coli* ATCC 25922 was used as a control for the antimicrobial susceptibility testing.

### Bacterial Growth Curves

Growth curves for O4:KUT2, O4:K9, O4:K8 and O3:K6 (four strains per serotype) were performed as described previously (Aroori et al., 2013). Strains were cultured at 37°C in 3% NaCl of brain heart infusion (BHI) broth with pH 7.4. A SpectraMax i3x microplate reader (Molecular Devices, United States) was used to measure the OD value at 600 nm for a total of 24 h (1 h interval). Three times were repeated.

### Phylogenetic Analyses

A phylogenetic tree was created by genomes of 16 O4:KUT2 strains and 1 O4:K9 strain sequenced in this study and 842 genomes downloaded from NCBI (Chen et al., 2020). The MUSCLE (Version: 3.8.31) software was used to compare multiple protein sequences, and the phylogenetic tree was constructed by TreeBeST (Version: 1.9.2) software using the neighbor joining (NJ) method.

### Ethics

This study was approved by the Ethics Committees of The First Affiliated Hospital, School of Medicine, Zhejiang University. All participants provided written informed consent.

## Results

### K Antigen Gene Clusters of O4:KUT2 Serotype Strain

**FIGURE 1:**
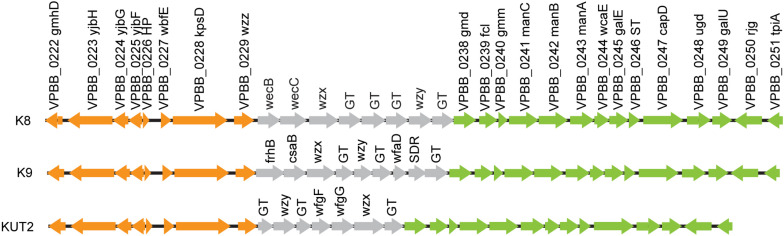
K antigen gene clusters of O4:KUT2 serotype strain. Orange and green genes are homologous. Gray represents different genes.

O4:KUT2 serotype was untypeable with K antisera, including K72, K73, K74, and K75 antisera. By BLAST K antigen gene clusters sequences of 16 whole genome sequenced O4:KUT2 serotype strains with 48 kinds of named K, we found that KUT2 antigen gene clusters sequences were specific and the highest identities to K9 and the second identities to K8. The main difference of the three K was that the genes between *wzz* and *gmd* were different (Figure 1).

### Prevalence of O4:KUT2 Strains

**FIGURE 2:**
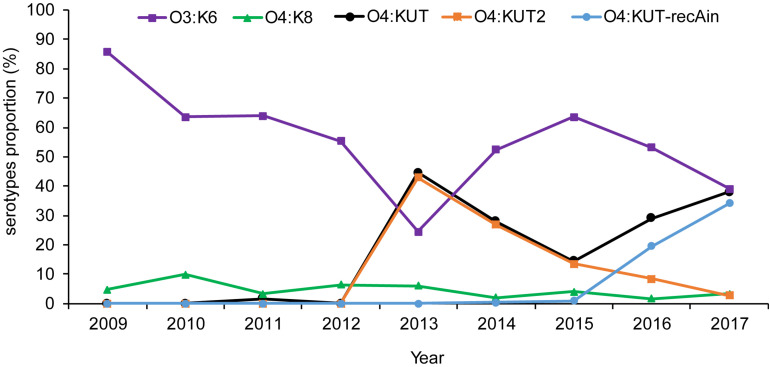
Distribution of the main *Vibrio parahaemolyticus* serotypes isolated from acute diarrheal patients in Zhejiang province between 2009 and 2017. O3:K6 was the strain agglutinating with O3 antisera and K6 antisera. O4:KUT was the strain agglutinating with O4 antisera and not agglutinating with all known K antisera including K72, K73, K74, and K75 antisera. O4:KUT2 was the positive strain of O4:KUT2 screening PCR. O4:KUT-recAin was the positive strain of O4:KUT-recAin screening PCR and agglutinating with O4:KUT-recAin strain-specific K antisera (Chen et al., 2020). O4:KUT includes O4:KUT2 and O4:KUT-recAin.

Based on the serotypes obtained by PCR screening and WGS were consistent in O4:KUT-recAin strains, we have not sequenced more O4:KUT2 strains. The prevalence of O4:KUT2 strains were based on O4:KUT2 screening PCR results. Retrospective analysis by O4:KUT2 screening PCR showed that there were 207 O4:KUT2 serotype strains among 1870 strains of *V. parahaemolyticus* isolated from acute diarrheal patients in Zhejiang province from 2009 to 2017. O4:KUT2 serotype appeared in 2013 and was the most dominant serotype in that year, as 43% (86/200) of *V. parahaemolyticus* were O4:KUT2 serotype, while 24.5% (49/200) were O3:K6 serotype (Figure 2). O4:KUT strains were almost O4:KUT2 in 2013 (96.7%, 86/89), 2014 (96.5%, 55/57) and 2015 (93.1%, 27/29).

We also conducted surveys throughout Jiangsu and Liaoning province. There were 73 O4:KUT2 serotype strains among 516 strains of *V. parahaemolyticus* in Jiangsu province from 2014 to 2017, and 15 O4:KUT2 serotype strains among 442 strains of *V. parahaemolyticus* in Liaoning province from 2012 to 2017. The earliest detection time in Liaoning province was also 2013.

### The Virulence-Associated Genes of O4:KUT2 Serotype Strains

All 295 O4:KUT2 serotype strains were postitive for *tlh* and *tdh* genes, and negative for *trh* gene. No O4:KUT2 serotype strain had the unique *toxRS* sequence detectable by GS-PCR. Therefore O4:KUT2 serotype strains had the characteristics as follow: *tlh*^+^ *tdh*^+^ *trh*^–^ *toxRS/*new^–^, which were not belongs to pandemic clones. O4:KUT2 serotype strains did not present *orf8* gene. T3SS1 genes and T3SS2α genes were detected in all O4:KUT2 serotype starins, while no T3SS2β genes were detected.

### Antimicrobial Susceptibility

About 91.5% O4:KUT2 serotype strains were resistant to ampicillin and 100% strains were sensitive to ampicillin-sulbactam, piperacillin-tazobactam, meropenem, imipenem, levofloxacin, trimethoprim-sulfamethoxazole, and tetracycline antibiotics. The resistance rate to cefazolin was 5.5%. High rate of O4:KUT2 serotype strains exhibited intermediate levels of susceptibility to cefuroxime (40%) and amikacin (17%). The sensitivity rate to piperacillin, ceftazidime, cefotaxime, cefoxitin, gentamicin, ciprofloxacin, and chloramphenicol were 97.5, 99, 93, 98, 99, 98, and 99.5%, respectively.

### The Growth Curves of O4:KUT2 Serotype Strains

**FIGURE 3:**
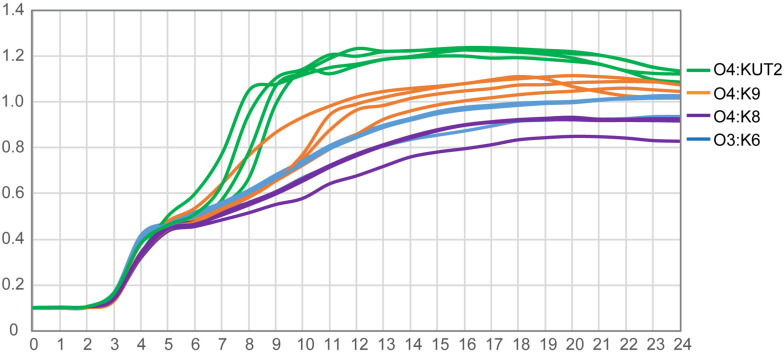
The growth curves of O4:KUT2, O4:K9, O4:K8, and O3:K6 serotype. Four strains of each serotype were cultured at 37°C in 3% NaCl of BHI broth with pH 7.4. Three times were repeated.

The growth curves of O4:KUT2 strains were different with O4:K9, O4:K8, and O3:K6 serotype strains incubated at 37°C in 3% NaCl of BHI broth with pH 7.4. Under the same nutrition condition, the growth restriction of O4:KUT2 strains affected by the concentration was smaller than O4:K9, O4:K8, and O3:K6 serotype strains, while the concentration of bacteria entering the stable period was higher (Figure 3).

### Multilocus Sequence Typing Analysis and Large Fragment Insertions at the *recA*

Multilocus sequence typing analysis of 35 O4:KUT2 strains isolated from Zhejiang province showed that all of them were ST332 except one. After whole-genome analysis of this particular strain, a large fragment inserted at *recA*141 was found which was the allele gene of *recA* in ST332 at nucleotide position 497, and the inserted genes contained a *recA*276 allele gene. We named this strain “O4:KUT2-recAin-1.” Two other insertions were detected at the same positon at *recA*141 by whole genome sequencing of sixteen O4:KUT2 strains (3 from Liaoning province, 3 from Jiangsu province and 10 from Zhejiang province), named “O4:KUT2-recAin-2” and “O4:KUT2-recAin-3,” respectively. In the 16 sequenced O4:KUT2 strains, there were one O4:KUT2-recAin-2 strain isolated from Jiangsu province in 2014 and one O4:KUT2-recAin-3 strains isolated from Zhejiang province in 2017, while there were two O4:KUT2-recAin-1, one isolated from Jiangsu province in 2015 and one isolated from Zhejiang province in 2017 (Table 1).

**TABLE 1:** The *recA* insertion and ST of the sixteen sequenced O4:KUT2 strains.

**Strains**	**Year**	**Area**	**O**	**K**	**recA insertion**	**ST**
42	2013	Zhejiang	4	KUT	O4:KUT2	332
365	2013	Zhejiang	4	KUT	O4:KUT2	332
542	2014	Zhejiang	4	KUT	O4:KUT2	332
103	2015	Zhejiang	4	KUT	O4:KUT2	332
595	2015	Zhejiang	4	KUT	O4:KUT2	332
168	2016	Zhejiang	4	KUT	O4:KUT2	332
761	2016	Zhejiang	4	KUT	O4:KUT2	332
2502	2017	Zhejiang	4	KUT	O4:KUT2	332
856	2017	Zhejiang	4	KUT	O4:KUT2-recAin-3	332
2571	2017	Zhejiang	4	KUT	O4:KUT2-recAin-1	1844
976	2014	Jiangsu	4	KUT	O4:KUT2-recAin-2	no ST
1228	2015	Jiangsu	4	KUT	O4:KUT2-recAin-1	1844
1048	2016	Jiangsu	4	KUT	O4:KUT2	332
3002	2013	Liaoning	4	KUT	O4:KUT2	332
3024	2014	Liaoning	4	KUT	O4:KUT2	332
3156	2015	Liaoning	4	KUT	O4:KUT2	332

Core genome multilocus sequence typing analysis of whole genomes of sixteen sequenced O4:KUT2 strains showed that they had no different alleles at 2242 loci and no data for other 12 loci. These results indicated that these strains from different years and different areas were come from the same ancestor, with different inserts at *recA*.

### Clinical Presentation of Patients Positive for *V. parahaemolyticus* O4:KUT2

Clinical data from O4:KUT2 infected patients were summarized in Table 2. The age of patients ranged from 1 month to 83 years old, the median age was 32 years old. There was no difference in infection rate between men and women. Leukocytes and proportion of neutrophils were higher in the O4:KUT2 infected patients than in normal persons. 39.3% (64/163) of O4:KUT2 infected patients had leukocytes in the stool and 25.2% (41/163) had red blood cells in the stool. 57.2% (95/166) O4:KUT2 infected patients exhibited watery stools and median frequency of diarrhea was five times per day.

**TABLE 2:** Demographic and clinical characteristics and laboratory test values of patients positive for *Vibrio parahaemolyticus* O4:KUT2 at first presentation.

**Variable**	**Normal range**	**O4:KUT2 (n = 201)**
Median age (range)	–	32 (1 month–83 years)
Male no./total no. (%)	–	102/201 (50.75)
Leukocytes mean ± SD × 10^–9^/L/total no.	4.0–10.0	12.6 ± 4.5/120
Proportion of neutrophils mean ± SD%/total no.	50.0–70.0	81.7 ± 10.8/120
Proportion of lymphocytes mean ± SD%/total no.	20.0–40.0	12.9 ± 9.5/120
Hemoglobin mean ± SD g/L/total no.	131–172	142.2 ± 19.3/120
Platelets mean ± SD × 10^–9^/L/total no.	83–303	217.3 ± 60.6/120
*Diarrhea*		
Median frequency, times/day	–	5
Watery stool no./total no. (%)	–	95/166 (57.2)
Loose stool no./total no. (%)	–	58/166 (34.9)
Leukocytes HPF no./total no. (%)	0	64/163 (39.3)
Red Blood Cell HPF no./total no. (%)	0	41/163 (25.2)

## Discussion

*Vibrio parahaemolyticus*, a halophilic Gram-negative bacterium, is one of the major diarrheal pathogen in coastal area. It can be classified according to the serotypes of heat stable O antigen and heat unstable K antigen. So far, there are 11 named O-antigen serotypes and 69 named K-antigen serotypes of *V. parahaemolyticus.* O4:KUT2 serotype was untypeable with K antisera, including K72, K73, K74, and K75 antisera, indicated that it was a new K serotype. Retrospective analysis showed that O4:KUT2 serotype strains were first appeared in 2013 and was the most dominant serotype in that year in Zhejiang province. These results made us very curious about why it became a serotype with an infection rate of more than O3:K6 and how O4:KUT2 serotype appeared.

**FIGURE 4:**
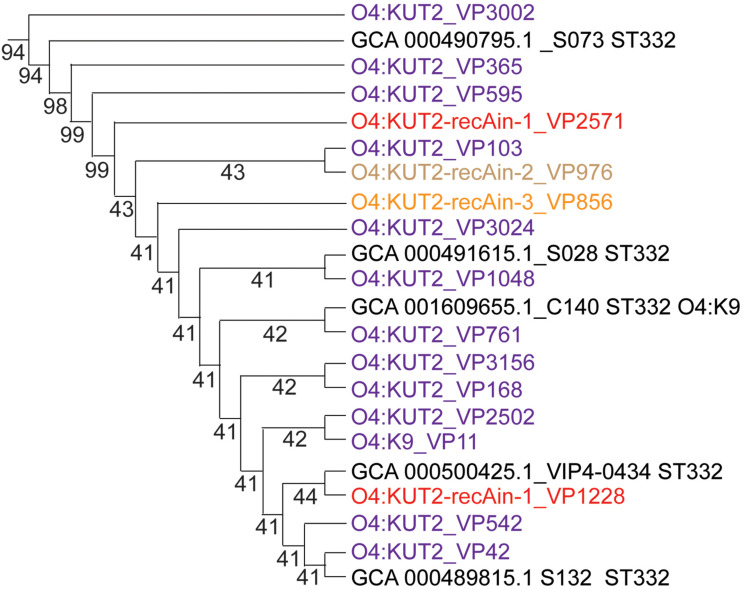
The phylogenetic branch of O4:KUT2 strains. The whole phylogenetic tree (not shown) was created by genomes of 16 O4:KUT2 strains and 1 O4:K9 strain sequenced in this study and 842 genomes downloaded from NCBI (Chen et al., 2020). The MUSCLE (Version: 3.8.31) software was used to compare multiple protein sequences, and the phylogenetic tree was constructed by TreeBeST (Version: 1.9.2) software using the neighbor joining (NJ) method. O4:KUT2 strains were all concentrated on the same branch. The genomes downloaded in NCBI was marked black.

Analysis of the K antigen gene clusters of O4:KUT2 serotype strain showed that they were the highest identities to K9 and the second identities to K8. Seven O4:K9 strains isolated from Zhejiang province were all ST332, the same ST as O4:KUT2 strains. Compared the whole genomes of O4:K9 strains from acute diarrhea patients in Zhejiang province in 2012 with O4:KUT2 strains, they had no different alleles at 2249 loci and no data for other 12 loci, including MLST and cgMLST loci. A phylogenetic tree was created by genomes of 16 O4:KUT2 strains and 1 O4:K9 strain sequenced in this study and 842 genomes downloaded from NCBI. 16 O4:KUT2 strains were concentrated on the same branch (Figure 4). The other strains on this branch were all ST332 and had no insertion at *recA* gene. C140 strain was O4:K9 serotype from in the BioSample SAMN04378285. Although S073 strain was O3:K6 serotype from in the BioSample SAMN02338930, S073, S028, S132, VIP4-0434, and C140 strains had the same K antigen gene clusters which belonged to K9. The serotype of S073 strain should be rechecked. Thus O4:KUT2 strains might be derived from O4:K9 strains.

O4:KUT2 strains were emerged in 2013. Then O4:KUT2 with a large fragment inserted at *recA* strains were appeared. The insertion of a large fragment at the *recA* locus was first reported in *Vibrio cholerae* (non-O1/O139 strain, isolated from water in 2009) in 2015 (Rapa et al., 2015). In the same year, a *V. parahaemolyticus* O4:K8 strain (090-96 strain, isolated from *Homo sapiens* in 1996) was also found an insertion at the *recA* locus (González-Escalona et al., 2015). Our group reported a new K serotype of *V. parahaemolyticus* strain named “O4:KUT-recAin,” which had the same inserted fragment as in the 090-96 strain, but most genes were different from those in *V. cholera* (Chen et al., 2020). In addition, MAVP-R, S130, and S134 strains were reported to have been inserted at *recA* (Xu et al., 2017). The insertion of a large fragment at the *recA* in O4:KUT2 strains had three kinds which were different from reports before. Different insertions indicated that these inserts were independent events, although they all inserted at 497bp of *recA* gene and repeated the sequence GTCTCCA at the end insertion (Table 3).

**TABLE 3:** The strains with different large fragment insertions at the *recA*.

**Strains**	**Original *recA* allele**	**New *recA* allele**	**Insertion side at *recA***	**Repeat sequence**
O4:KUT2-recAin-1; HZ17-098; HZ17-221	141	276	497	GTCTCCA
O4:KUT2-recAin-2	141	120	497	GTCTCCA
O4:KUT2-recAin-3; HZ17-125; HZ17-129	141	None	497	GTCTCCA
MAVP-R; S130; S134	21	107	497	GTCTCCA
090-96; O4:KUT-recAin	3	107	497	GTCTCCA

In *V. parahaemolyticus* genome assembly and annotation report data^2^, there were two O4:KUT2-recAin-1 (HZ17-098 and HZ17-221) and two O4:KUT2-recAin-3 (HZ17-125 and HZ17-129) starins from diarrheal patients in Hangzhou in 2017. In addition, two strains isolated from marine organisms had the same insertion as O4:KUT2-recAin-3 at *recA*, but their serotypes were O5:K15 (S020, 1994) and O4:K42 (GIMxtf283-2012.12, 2012), not O4:KUT2. No identical insertion was found in other sequenced strains of *Vibrionaceae* family. Multilocus sequence typing analysis of S020 strain and GIMxtf283-2012.12 strain showed that they were different from O4:KUT2-recAin-3 strains. So we could conclude that O4:KUT2-recAin-3 was not serotype transformed by S020 or GIMxtf283-2012.12 strains.

Therefore, we speculated that the emergence of O4:KUT2 was the result of seroconversion of O4:K9 and the large fragment insertions at the *recA* were independent insert events. Further studies will be focused on the molecular mechanism of serum transformation and insertion.

In addition, we observed that the growth curve of O4:KUT2 was closer to O4:K9 compared with O4:K8 and O3:K6. The growth rate of O4:KUT2 was slightly higher than O3:K6, which may related to its prevalence. However, after its prevalence in 2013, O4:KUT2 strains gradually decreased from 2014 to 2017. This phenomenon may be due to O4:KUT2 strains not belonging to pandemic clones which lack of unique *toxRS* sequence detectable by GS-PCR. Although the clinical symptoms of O4:KUT2 patients were lighter than those of O3: K6 group (Chen et al., 2020), its pathogenicity was consistent with the presence of *tdh* gene and T3SS genes.

## Data Availability Statement

The datasets generated for this study can be found in the NCBI under Bioproject number PRJNA644273 (https://www.ncbi.nlm.nih.gov/bioproject/PRJNA644273).

## Ethics Statement

The studies involving human participants were reviewed and approved by the Ethics Committees of The First Affiliated Hospital, School of Medicine, Zhejiang University. The patients/participants provided their written informed consent to participate in this study.

## Author Contributions

QZ designed the assays, analyzed the data, and wrote the manuscript. XC designed the study and collected the strains. YL and RW collected the strains, serotyped the strains, detected the virulence-associated genes, and did antimicrobial susceptibility testing. JC collected the strains. YC designed the study. All authors contributed to the article and approved the submitted version.

## Conflict of Interest

The authors declare that the research was conducted in the absence of any commercial or financial relationships that could be construed as a potential conflict of interest.

## Footnotes

1 https://pubmlst.org/vparahaemolyticus/

2 https://www.ncbi.nlm.nih.gov/genome/browse/#!/prokaryotes/691/, 11/06/2019
