# Supplementary material for: Virulence, Antimicrobial Susceptibility, Molecular and Epidemiological Characteristics of a New Serotype of Vibrio parahaemolyticus From Diarrhea Patients
Source: Front Microbiol. 2020 Aug 21;11:2025. doi: 10.3389/fmicb.2020.02025 (PMC7475705; doi:10.3389/fmicb.2020.02025)
Supplement: Supplementary file 2 [file Table_2.DOCX]

Table S1. Primers for detected genes.

| **gene** | **Forward primer** | **Reverse primer** | **Length (bp)** | **Annealing temperature** |
| --- | --- | --- | --- | --- |
| for O4:KUT2 strains | CTCCGTCAGAGCGTAATC | TCGTCTCAAAGCGTAAAA | 441 | 55℃ |
| recA | GAAACCATTTCAACGGGTTC | CCATTGTAGCTGTACCAAGCACCC | 773 | 58℃ |
| gyrB | GAAGGBGGTATTCAAGC | GAGTCACCCTCCACWATGTA | 629 | 58℃ |
| dnaE | CGRATMACCGCTTTCGCCG | GAKATGTGTGAGCTGTTTGC | 596 | 58℃ |
| dtdS | TGGCCATAACGACATTCTGA | GAGCACCAACGTGTTTAGC | 497 | 58℃ |
| pntA | ACGGCTACGCAAAAGAAATG | TTGAGGCTGAGCCGATACTT | 470 | 58℃ |
| pyrC | AGCAACCGGTAAAATTGTCG | CAGTGTAAGAACCGGCACAA | 533 | 58℃ |
| tnaA | TGTACGAAATTGCCACCAAA | AATATTTTCGCCGCATCAAC | 463 | 58℃ |
| tlh | AAAGCGGATTATGCAGAAGCACTG | GCTACTTTCTAGCATTTTCTCTGC | 450 | 58℃ |
| trh | TTGGCTTCGATATTTTCAGTATCT | CATAACAAACATATGCCCATTTCCG | 500 | 54℃ |
| tdh | GTAAAGGTCTCTGACTTTTGGAC | TGGAATAGAACCTTCATCTTCACC | 269 | 54℃ |
| orf8 | GTTCGCATACAGTTGAGG | AAGTACAGCAGGAGTGAG | 746 | 57℃ |
| toxRS/new | TAATGAGGTAGAAACA | ACGTAACGGGCCTACA | 651 | 45℃ |
| VP1670 (vscP) | ACCGATTACTCAAGGCGATG | TACGTTGTTGGCGTGATTGT | 392 | 60℃ |
| VP1686 (copS) | CAAAAGCGATCACAAAAGCA | AGCGACTTAACGGCATCATC | 283 | 60℃ |
| VP1689 (vscK) | AAGGTTGGCAAAAAGCGTTA | GCTCTTCAACGAGCCAAGAG | 192 | 60℃ |
| VP1694 (vscF) | ACGATGCGACCAACAGTGTA | TTTTAATTGCATCGGTGACG | 96 | 60℃ |
| VP1362 (vopB2) | CTGCAGGTATCGCATCTTCA | TTAGAACCAACCGACGAAGC | 343 | 60℃ |
| VP1339 (vscC2) | GATTCGCGGAACTCAAGAAG | CTTGTCCGAGATCAACGTCA | 250 | 60℃ |
| VP1335 (vscS2) | ATGTAACGGCGGCTAGCTTA | CAAACTGTGTCAGTAGCACCA | 174 | 60℃ |
| VP1327 (vopT) | TGGCGAAAGAGCCATTAGAT | TCAACTCCAAATTCGCCTTC | 97 | 60℃ |
| vscC2 | GTACTTTGCTGTCTAACC | CTTACTCTTAACTTCCGACG | 1400 | 60℃ |
| vopB2 | GAGCCTGTTGCTCTATGGAGCCAGG | CGACACAGAACGCAATGCTTGCTCG | 942 | 60℃ |
| vopC | AACCAACTTGCGACTAAATC | TCCCGACAGTTTTTCTGCAC | 594 | 60℃ |
| vscS2 | TTGATGTTGTTTCGGCTAGC | CCACCGCCGAACTCGGCTAACAAG | 224 | 60℃ |
